# Gender-Based Morphometric Analysis of the Distal Femur in Patients Undergoing Total Knee Arthroplasty

**DOI:** 10.7759/cureus.104641

**Published:** 2026-03-03

**Authors:** Jagdeep S Rehncy, Amandeep S Bakshi, Pranav Bansal, Harjit Kanwar S Chawla

**Affiliations:** 1 Orthopaedics, Government Medical College and Rajindra Hospital, Patiala, Patiala, IND

**Keywords:** distal femur morphology, implant mismatch, indian population, sex differences, total knee arthroplasty

## Abstract

Background

Total knee arthroplasty (TKA) is the definitive treatment for end-stage knee osteoarthritis; however, prosthesis-bone mismatch remains common and may compromise functional outcomes. Variations in distal femoral morphology related to sex and ethnicity contribute significantly to implant mismatch, particularly in Asian populations, where available implants are largely based on Caucasian anatomical data.

Objective

This study aimed to assess sex-based differences in distal femoral morphology in Indian patients undergoing TKA by evaluating specific intraoperative morphometric parameters: mediolateral (ML) width, anteroposterior (AP) length, medial and lateral AP lengths, intercondylar (IC) distance, and ML/AP aspect ratio. A secondary objective was to analyze the correlation between AP and ML dimensions to determine their utility in guiding implant sizing and minimizing prosthesis mismatch.

Methods

This prospective observational study included 50 patients undergoing primary TKA at a tertiary care center over one year. Intraoperative measurements of distal femoral dimensions, including ML width, AP length, medial and lateral AP lengths, and IC distance, were obtained using a vernier caliper. Aspect ratios (ML/AP×100) were calculated. Statistical analysis was performed using IBM SPSS Statistics for Windows, Version 20.0 (IBM Corp., Armonk, New York, United States), and sex-based comparisons and regression analyses were conducted.

Results

The mean age of patients was 48.98 years, with females constituting 80% of the cohort. The mean ML and AP dimensions were 66.06 mm and 58.64 mm, respectively, with a mean ML/AP ratio of 1.13. All measured parameters were significantly larger in males compared with females (p<0.05). The ML/AP ratio was also significantly higher in males (1.18 vs. 1.09). Regression analysis demonstrated a moderate correlation between AP length and ML width in females (R²=0.255), males (R²=0.495), and the combined cohort (R²=0.407), indicating that AP dimension alone is an incomplete predictor of ML width.

Conclusion

Significant sex-based differences exist in distal femoral morphology among Indian patients undergoing TKA. The moderate correlation between AP and ML dimensions suggests that reliance on AP-based implant sizing alone may result in prosthesis mismatch. These findings emphasize the need for population- and sex-specific implant designs to optimize surgical outcomes in TKA.

## Introduction

Total knee arthroplasty (TKA) remains the most effective and widely accepted surgical option for end-stage knee osteoarthritis when conservative treatment fails [1-3]. Although TKA reliably improves pain, limb alignment, and mobility, up to 40% of patients report unmet expectations following surgery [4]. Achieving a precise fit between the implant and resected bone is critical, as gaps at the bone-implant interface can increase technical difficulty, raise the risk of complications, and compromise long-term durability. Proper sizing and positioning of the femoral component are essential to avoid imbalance, patellar malalignment, and soft-tissue irritation; yet 28-68% of TKAs still demonstrate prosthesis mismatch despite the increasing number of available implant designs [5].

Both mediolateral (ML) and anteroposterior (AP) sizing inaccuracies have major clinical consequences. Oversizing in the ML dimension may result in soft-tissue irritation, while undersizing exposes cancellous bone and may increase postoperative bleeding [6]. AP mismatch may disturb flexion-extension gap balance, and anterior notching increases the risk of periprosthetic fracture [6,7]. Because AP-based sizing does not always correspond to ML morphology, femoral component selection may lead to a perfect fit, overhang, or underhang [8]. Overhang is particularly common, reported in up to 66% of femurs, and is associated with pain, reduced flexion, and poor outcomes; underhang, on the other hand, can elevate cancellous bone stress and predispose to early loosening [8,9]. Rotational mismatch adds further challenges, including patellofemoral incongruence, anterior knee pain, and accelerated polyethylene wear [10].

Optimal implant selection depends on accurate morphometric data, including ML width, AP length, patellar dimensions, and side-specific aspect ratios, all of which vary by sex and ethnicity [11]. Most prostheses used in Asian populations are adapted from Caucasian anatomical models and may not match regional morphology [8]. Given the limited anthropometric data available for the Indian population, particularly regarding distal femur and proximal tibia dimensions, it is essential to assess intraoperative anatomy to guide appropriate implant design and selection [12].

Therefore, the primary objective of this study was to evaluate sex-based differences in distal femoral morphology by measuring specific morphometric parameters, including ML width, AP length, medial and lateral AP lengths, intercondylar (IC) distance, and ML/AP aspect ratio. The secondary objective was to analyze the correlation between AP and ML dimensions to assess the utility of AP-based scaling in predicting ML fit and to evaluate the implications of these relationships for implant sizing and prosthesis mismatch in TKA.

## Materials and methods

This prospective study was carried out in the Department of Orthopaedics, Government Medical College and Rajindra Hospital, Patiala, India, after obtaining approval from the institute's Institutional Ethics Committee (approval number: 2024/23608-675) and written informed consent from all participants. Data were collected from inpatients over a one-year period, and a total of 50 individuals undergoing TKA were enrolled sequentially based on the predefined inclusion and exclusion criteria.

Inclusion criteria included adult patients over 18 years of age with a diagnosis of primary or secondary knee osteoarthritis. Exclusion criteria included patients with congenital or acquired knee deformities, prior trauma, significant bone loss requiring augmentation, anterior notching, or severe flexion gap laxity after the distal femoral cut or those unwilling to participate.

Consecutive sampling was used to enroll patients. In this prospective TKA cohort, all eligible patients were included in sequence as they presented, a method commonly employed in clinical orthopedic research. Patient evaluation included a detailed clinical history, relevant investigations, and assessment of medical background.

The sample size was determined by feasibility. The study's target sample (n=50) reflected the number of patients realistically available during the enrollment period at a single tertiary care center, rather than an a priori statistical calculation due to limited resources and time. No formal sample size or power calculation was performed. While this approach is common in observational morphometric studies, we acknowledge that the absence of an a priori power analysis may limit the statistical strength of subgroup comparisons, particularly those based on sex. These findings should therefore be interpreted as exploratory, and future research with larger, powered cohorts is warranted to validate the observed morphometric differences.

Measurement technique

All measurements were performed intraoperatively using a digital vernier caliper with an accuracy of 0.01 mm. After completing standard soft-tissue release and osteophyte removal, the distal femoral cuts were made using an intramedullary alignment guide. Morphometric measurements were taken immediately after the distal femoral bone cut, with the knee flexed to 90 degrees to ensure consistent exposure and positioning. The ML width was measured at the widest medial-to-lateral condylar distance, while the AP length was recorded from the deepest point of the anterior trochlear groove to the posterior condyles. Medial and lateral AP lengths were measured from the anterior cortex to the posterior condylar margin on the respective sides. IC distance was defined as the width of the IC notch at its narrowest point. Femoral rotational alignment was standardized using the posterior condylar axis as the reference. All measurements were performed by a single experienced orthopedic surgeon to reduce interobserver variability. However, no formal intraobserver reliability testing or repeated measurements were conducted, which is acknowledged as a study limitation.

All statistical analyses were performed using IBM SPSS Statistics for Windows, Version 20.0 (IBM Corp., Armonk, New York, United States). Continuous variables (e.g., ML width, AP length, aspect ratios) were tested for normality using the Shapiro-Wilk test. Data that followed a normal distribution are presented as mean±standard deviation (SD), while non-normally distributed variables are reported as median and interquartile range (IQR).

Comparisons between male and female patients were made using the independent-samples t-test for normally distributed continuous variables and the Mann-Whitney U test for non-normally distributed variables. Categorical variables were compared using the chi-squared test or Fisher's exact test where appropriate. A p-value of less than 0.05 was considered statistically significant.

No randomization was involved in this observational study; therefore, intention-to-treat analysis was not applicable. All patients who met the inclusion criteria and completed intraoperative measurements were included in the final analysis.

## Results

Demographic characteristics

The mean age of participants was 48.98±6.72 years (range: 38-65). The overall mean BMI was 24.8±2.3 kg/m² (range: 21.2-29.6). Females accounted for the majority of the sample (80%; n=40), while males represented 20% (n=10). These values are summarized in Table 1.

**Table 1:** Demographic data

Variable	Mean±SD (range)	Percentage
Mean age	48.98±6.72 (38-65)	
Males	10	20
Females	40	80
Mean BMI (kg/m^2^)	24.8±2.3 (21.2-29.6)	

Overall morphometric measurements

The mean ML width was 66.06±1.58 mm, and the mean AP length was 58.64±1.69 mm. The mean ML/AP aspect ratio was 1.13±0.04. Additional parameters included the following: medial anteroposterior (MAP) length: 49.15±2.05 mm; lateral anteroposterior (LAP) length: 46.11±1.79 mm; and IC distance: 16.41±1.36 mm. These measurements are detailed in Table 2.

**Table 2:** Intraoperative measurements ML: mediolateral; AP: anteroposterior; MAP: medial anteroposterior; LAP: lateral anteroposterior; IC: intercondylar

Variable	Mean	SD
ML	66.06	1.58
AP	58.64	1.69
ML/AP	1.13	0.04
MAP	49.15	2.05
LAP	46.11	1.79
IC	16.41	1.36

Sex-based comparison of morphometry

Statistically significant sex-based differences (p<0.05) were observed across all morphometric parameters. Males demonstrated consistently larger mean values than females as follows: ML: 69.63±1.42 mm (males) vs. 63.19±1.07 mm (females) (t=13.12; p<0.001); AP: 61.58±1.34 mm vs. 55.72±1.25 mm (t=13.10; p<0.001); MAP: 53.36±2.07 mm vs. 45.98±1.85 mm (t=10.36; p=0.007); LAP: 49.52±1.65 mm vs. 42.13±1.48 mm (t=13.70; p<0.001); and IC: 19.85±1.23 mm vs. 13.22±1.03 mm (t=16.50; p=0.003). The ML/AP aspect ratio was also significantly higher in males (1.18±0.03) than in females (1.09±0.02), with a p-value of 0.001 (t=10.35; p=0.001). Sex-wise comparison data are provided in Table 3.

**Table 3:** Comparison of intraoperative measurements among males and females *: significant ML: mediolateral; AP: anteroposterior; MAP: medial anteroposterior; LAP: lateral anteroposterior; IC: intercondylar

Variable	Males	Females	P-value
ML	69.63	63.19	0.001*
AP	61.58	55.72	0.000*
ML/AP	1.18	1.09	0.001*
MAP	53.36	45.98	0.007*
LAP	49.52	42.13	0.000*
IC	19.85	13.22	0.003*

Regression analysis

**Figure 1:**
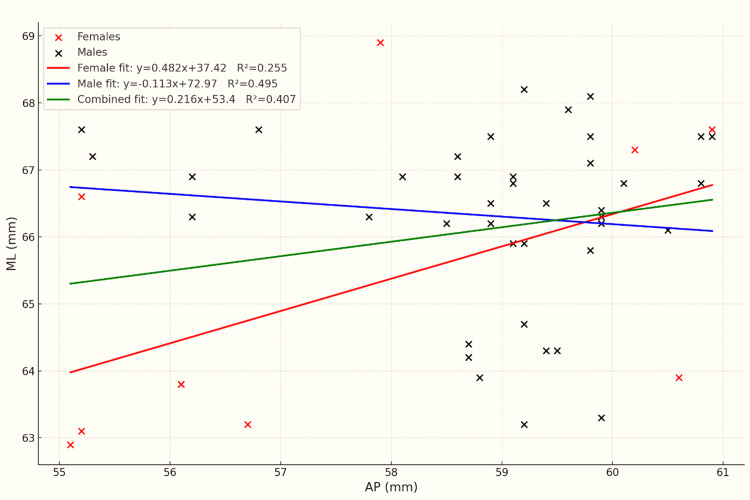
AP vs. ML regression for males, females, and combined data AP: anteroposterior; ML: mediolateral

Linear regression assessed the relationship between AP length and ML width. In the combined sample, linear regression analysis demonstrated a statistically significant positive association between AP and ML dimensions (ML=0.216×AP+53.40). The model was statistically significant (t=7.02; p<0.001) and explained 40.7% of the variance (R²=0.407). The 95% confidence interval for the slope ranged from 0.155 to 0.278. In males, the regression model showed a stronger association, explaining 49.5% of the variance (R²=0.495). The slope was statistically significant (t=5.54; p<0.001), with a 95% confidence interval ranging from 0.298 to 0.552. In females, the model explained 25.5% of the variance (R²=0.255). The slope was also statistically significant (t=3.89; p=0.001), and the 95% confidence interval ranged from 0.201 to 0.534. Assumptions of linearity, homoscedasticity, and normality of residuals were verified through residual plot analysis. The regression trends for males, females, and combined data are illustrated in Figure 1.

## Discussion

The average patient age and BMI in this study reflect a typical demographic of middle-aged individuals with normal to slightly overweight profiles undergoing TKA in India. The predominance of female participants is consistent with established epidemiologic trends showing a higher burden of advanced osteoarthritis among women.

Several investigations have examined femoral morphology relevant to TKA [11,12]. Much attention has focused on sex-specific implant designs, which are proposed to better match female knee anatomy [13], and many manufacturers have incorporated these findings into implant development. However, several earlier studies relied on small cadaver samples and predominantly white populations [14], limiting the generalizability of their conclusions to real-world surgical practice and to Asian populations. Moreover, growing evidence suggests that Asian knees differ substantially in size and morphology compared with those of white individuals. Singapore, being a multiethnic Asian nation, provides an appropriate and diverse population in which to evaluate these anatomical differences [15,16]. Hence, the present study was conducted to assess morphometric characteristics in patients undergoing TKA in a tertiary care center to improve implant matching and long-term surgical outcomes.

The results of our study demonstrated distinct and statistically significant differences in distal femoral morphology between males and females. Similar to previous reports, such as those by Hitt et al. [14] and Urabe et al. [17], our study also documented differential aspect ratios among men and women, indicating a proportionally wider distal femur in male patients. Within each sex, a notable range of ML/AP values was observed, consistent with prior findings that knee morphology varies considerably even within the same gender group [14,17].

The clinical implications of these variations have been well described. Hitt et al. [14] highlighted that ML overhang may lead to soft-tissue irritation and difficulty achieving balanced knee flexion, whereas Lonner et al. [18] reported that downsizing femoral components in women, who typically present with smaller AP dimensions, may result in anterior notching or excessive posterior condylar resection. Although some implant systems have introduced sex-specific designs based on such anthropometric studies, whether these implants translate into improved functional outcomes remains unclear, as noted by multiple authors [19-21].

In our dataset, females exhibited consistently smaller ML, AP, MAP, LAP, and IC measurements than males, reinforcing earlier findings that women tend to have smaller femoral dimensions. The statistically significant differences in ML/AP ratio between sexes indicate notable variation in distal femoral proportionality. This suggests that sex-specific morphometric differences may have relevance during implant selection, particularly to minimize ML overhang or underhang. While AP length showed a statistically significant association with ML width, the moderate R² value (0.407) indicates that AP alone may not be a reliable predictor of ML dimension. These findings highlight the potential benefit of independently measuring both parameters intraoperatively rather than relying solely on AP-based scaling during implant selection. Additionally, although regression coefficients were statistically significant, the moderate R² values highlight considerable inter-individual variation, particularly among female patients. This supports the idea that intraoperative sizing should assess both ML and AP parameters separately rather than assuming proportional scaling.

Our study, similar to that of Urabe et al. [17], was limited by an unequal representation of sexes because a higher proportion of TKA candidates were female. Another limitation concerns the minor measurement uncertainty inherent in manual caliper assessments, an issue also acknowledged by Lonner et al. [18]. Furthermore, while all measurements were performed by a single surgeon to reduce interobserver variability, no intraobserver reliability testing or repeated measurements were conducted, which may affect reproducibility in other settings. Nevertheless, the magnitude of differences observed between sexes far exceeded the expected measurement margin of error, supporting the overall robustness of our findings. The lack of a formal power calculation also limits inferential strength, particularly for sex-based subgroup comparisons. As in other orthopedic studies without a sample size calculation, this remains an important caveat and supports the need for larger, hypothesis-driven studies in the future.

Comparisons with anthropometric studies in other populations highlight important ethnic variation. Vaidya et al. [22] demonstrated that Indian knees differ significantly from white populations, while Yue et al. [23] and Ho et al. [24] showed that Chinese knees are generally smaller and have narrower aspect ratios than white knees. Our findings similarly revealed larger aspect ratios in both males and females compared with Western parameters reported by Lonner et al. [18], indicating proportionally narrower femora in our study population. This aligns with observations by Cheng et al. [25], who attributed the higher incidence of ML overhang in Asian patients to their narrower femoral morphology.

Collectively, these results highlight the potential value of population-specific and sex-specific implant design in TKA. However, we acknowledge that our study was observational, single-centered, and not powered to assess clinical outcomes. Therefore, while our findings are anatomically relevant and hypothesis-generating, definitive conclusions regarding implant performance or patient outcomes require validation in larger, multi-center studies with long-term follow-up.

## Conclusions

This study demonstrates significant sex-based differences in distal femoral morphology among patients undergoing TKA. Females showed smaller dimensions and lower ML/AP ratios compared to males, indicating differences in both size and shape. The moderate correlation between AP and ML dimensions suggests that AP-based sizing alone may be inadequate for achieving an optimal implant fit. These findings highlight anatomical variations in the Indian population compared with Western models and suggest that incorporating population- and sex-specific morphometric data could help reduce implant mismatch. However, as this was a single-center observational study without clinical outcome data, these conclusions should be considered hypothesis-generating. Future studies with larger, powered cohorts and clinical follow-up are needed to validate these implications and guide implant design.
